# Role of Antimicrobial Peptides in the Pathogenesis of Atopic Dermatitis

**DOI:** 10.1111/1346-8138.17975

**Published:** 2025-10-08

**Authors:** Ge Peng, Alafate Abudouwanli, Quan Sun, Yi Tan, Wanchen Zhao, Mengyao Yang, Shan Wang, Hideoki Ogawa, Ko Okumura, François Niyonsaba

**Affiliations:** ^1^ Atopy (Allergy) Research Center Juntendo University Graduate School of Medicine Tokyo Japan; ^2^ Department of Dermatology Beijing Children's Hospital, Capital Medical University, National Center for Children's Health Beijing China; ^3^ Faculty of International Liberal Arts Juntendo University Tokyo Japan

**Keywords:** antimicrobial peptide, atopic dermatitis, immune modulation, skin barrier, skin microbiota

## Abstract

Atopic dermatitis (AD) is a chronic inflammatory skin disorder characterized by barrier dysfunction, immune dysregulation, and microbial dysbiosis. Recent studies have highlighted the multifaceted roles of antimicrobial peptides (AMPs) both as innate defenders against microbial invasion and as regulators of immune responses and skin barrier homeostasis. This review synthesizes the current knowledge on the dysregulation of AMP expression in AD, the impact of Th2‐dominant inflammation on AMP‐mediated defense, and the complex relationship between AMP activity and the cutaneous microbiota (particularly in the context of *Staphylococcus aureus* colonization). We also explore the immunomodulatory and barrier‐stabilizing functions of AMPs, emphasizing their dual roles as both protective and potentially pathogenic agents depending on their expression levels and processing. Furthermore, emerging therapeutic strategies that aim to restore AMP function (such as vitamin D signaling, aryl hydrocarbon receptor activation, and synthetic AMPs) are discussed. A deeper understanding of AMP‐related mechanisms in AD may offer novel insights for precision‐targeted interventions that simultaneously address inflammation, barrier repair, and microbial imbalance.

## 1 Introduction

Atopic dermatitis (AD) is a common chronic inflammatory skin disease that affects up to 30% of children and 1%–10% of adults worldwide [1]. It often represents the initial manifestation of the “atopic march,” which is a progression toward other allergic conditions, such as asthma and allergic rhinitis [2, 3]. The pathogenesis of AD involves a complex interplay of genetic predisposition, immune dysregulation, and environmental factors [4]. One hallmark feature of this condition involves the disruption of the epidermal barrier, which increases transepidermal water loss and facilitates the penetration of allergens, irritants, and microbes. This scenario leads to intensely pruritic lesions and a self‐reinforcing itch‐scratch cycle, which substantially decreases quality of life [4]. Patients with AD exhibit increased susceptibility to cutaneous colonization or infection, particularly by *Staphylococcus aureus* and herpes simplex virus [5]. This microbial vulnerability is linked to both barrier dysfunction and innate immune defects. In addition, a dysregulated cytokine milieu is a critical factor in AD pathogenesis. Specifically, the overactivation of T helper type 2 (Th2) immune pathways, which is characterized by elevated interleukin (IL)‐4 and IL‐13 levels, leads to impaired barrier protein expression, defective antimicrobial peptide (AMP) production, and chronic inflammation [4, 6, 7]. Currently, AD is diagnosed via symptom observation and physical examination findings, with currently employed treatments aiming to address immune dysregulation, repair the skin barrier, and manage the associated symptoms [4].

The skin functions as a critical frontline barrier against environmental insults and microbial invasion, thus maintaining local and systemic homeostasis [8]. This barrier comprises two main components: a physical barrier that is predominantly formed by keratinocytes and a chemical barrier that includes lipids, enzymes, and AMPs [9]. Keratinocytes not only provide structural integrity through tight junctions and cornified envelope proteins but also act as immune‐competent cells by recognizing microbial threats and initiating innate immune responses [10]. Innate immunity in the skin is rapid and nonspecific; moreover, it is mediated by pattern recognition receptors (PRRs), such as toll‐like receptors (TLRs) and NOD‐like receptors, which are located on keratinocytes, dendritic cells, and macrophages. These receptors detect pathogen‐associated molecular patterns (PAMPs) and trigger the production of cytokines, chemokines, and AMPs, which recruit immune cells and initiate antimicrobial defense [11]. For example, the chemokine CXC motif chemokine ligand 14, which is produced by epidermal keratinocytes, exhibits circadian rhythmicity and can bind bacterial DNA from *S. aureus* , thereby activating TLR9‐dependent responses in dendritic cells and macrophages [12].

AMPs are small, cationic molecules that constitute a core component of cutaneous innate immunity [13]. AMPs act not only by directly eliminating a wide range of pathogens (including bacteria, fungi, and viruses) but also by modulating immune responses, influencing wound repair, and improving skin barrier integrity [13, 14]. Cathelicidins (particularly LL‐37 in humans) illustrate the dual functionality of AMPs. Specifically, these peptides exhibit potent antimicrobial activity while simultaneously promoting cytokine release, angiogenesis, and re‐epithelialization (functions that are critical for restoring tissue integrity after injury) [13, 14]. In AD, cathelicidin expression is frequently suppressed, thus weakening the skin's defense and skin barrier and contributing to increased susceptibility to microbial colonization [14]. AMPs also help to maintain microbial homeostasis by selectively inhibiting pathogenic species while preserving beneficial commensals, such as coagulase‐negative *Staphylococcus* (CoNS) strains, the loss of which may exacerbate dysbiosis and disease severity in AD [15]. In addition to their natural functions, AMPs are currently being explored as therapeutic agents. Synthetic AMPs have demonstrated promise in preclinical models because of their broad‐spectrum activity, resistance to microbial resistance mechanisms, and ability to promote wound healing [16, 17]. However, clinical translation is still limited by challenges such as proteolytic degradation in the skin and potential cytotoxicity at high concentrations [18, 19]. Ongoing research aims to optimize AMP stability, delivery, and immunological effects for future use in treating inflammatory skin diseases such as AD.

In this review, we focus on the multifaceted roles of AMPs in the pathogenesis of AD, including their dysregulated expression, effects on the skin microbiota and immune responses; genetic and epigenetic regulation; and therapeutic potential. By integrating recent findings, we aim to provide a comprehensive overview of how AMPs contribute to both skin defense and disease progression in AD and to explore future directions for AMP‐based interventions.

## 2 Dysregulation of AMPs in AD

### 2.1 Altered Expression of AMPs in AD Lesions

Compared with that in healthy individuals, the expression of AMPs in the skin tissues of patients with AD and other inflammatory skin diseases, such as psoriasis, is markedly altered. Early studies have demonstrated that the levels of LL‐37 and human β‐defensin (hBD)‐2 are significantly lower in AD skin than in psoriatic lesions, suggesting that AMP deficiency may contribute to increased microbial colonization in AD [20]. Similarly, the level of dermcidin‐1 (an AMP secreted by sweat glands) has also been reported to decrease in AD, thus further supporting the notion of compromised cutaneous defense [21]. However, AMP dysregulation in AD is not uniform. The levels of several AMPs, such as S100A7 and RNase 7, have been observed to be elevated in lesional AD skin [22]. Moreover, although constitutive hBD‐3 levels in keratinocytes are comparable between healthy individuals and AD patients, the functional activity of hBD‐3 against *S. aureus* is impaired under Th2‐dominant conditions [23]. Collectively, these findings indicate that the dysregulation of AMPs in AD is selective rather than generalized. Moreover, some AMPs are downregulated, others remain unchanged, and a few may even be upregulated. This imbalance disrupts the antimicrobial environment of the skin, weakens the barrier to infection, and may promote dysbiosis. The variability in AMP expression also appears to be influenced by disease severity and the local immune context, particularly regarding the cytokine milieu.

### 2.2 Th2 Cytokine‐Mediated Suppression of AMP Production

A major contributor to AMP dysregulation in AD is the Th2‐dominated immune response, which is specifically driven by elevated levels of IL‐4 and IL‐13. These cytokines directly decrease the expression of several critical AMPs (including LL‐37, hBD‐2, and hBD‐3), thereby impairing the skin's innate antimicrobial defense and increasing susceptibility to infections, most notably involving colonization by *S. aureus* [23, 24, 25]. Recent studies have demonstrated that this suppression is intricately linked to alterations in the vitamin D signaling pathway, which plays a crucial role in maintaining AMP production and immune homeostasis in the skin. In healthy skin, the bioactive form of vitamin D, calcitriol, binds to the intracellular vitamin D receptor (VDR), thus forming a complex that regulates gene expression by interacting with vitamin D response elements in target gene promoters, including those encoding AMPs such as *CAMP* (cathelicidin) and *DEFB4* (hBD‐2). This activation leads to the transcriptional induction of AMPs and strengthens the antimicrobial barrier of the skin. However, in AD, VDR expression is often downregulated in lesional keratinocytes, and serum vitamin D levels are frequently reduced [26], both of which may compromise this protective pathway. Moreover, IL‐4 and IL‐13 directly interfere with VDR‐mediated transcription. These cytokines can reduce VDR expression and impair its nuclear translocation and DNA‐binding capacity, thereby attenuating the vitamin D‐dependent induction of AMP genes [27, 28]. This effect not only weakens the direct antimicrobial response but also diminishes the immunomodulatory feedback loop that normally limits Th2 cytokine production.

In contrast, calcitriol counteracts this Th2‐driven suppression through a multilevel regulatory mechanism. Specifically, it modulates the VDR/GATA3/Gfi1 axis in Th2 cells, whereby it downregulates GATA3 and promotes the recruitment of the transcriptional repressor Gfi1 to the *Il13* promoter. It also facilitates VDR binding to the shared enhancer regions of *Il4* and *Il13*, thereby displacing GATA3 and enhancing histone deacetylase 1 recruitment, which leads to chromatin remodeling and transcriptional silencing of these cytokines [29, 30]. Interestingly, although Gfi1 typically suppresses *Il13* promoter activity, calcitriol further augments its suppressive function, thus reinforcing the downregulation of IL‐4 and IL‐13 expression [29]. This scenario creates a regulatory loop in which vitamin D signaling not only directly increases AMP expression but also reduces the cytokine milieu that otherwise inhibits its induction.

Taken together, these findings suggest that the restoration of vitamin D signaling (either via supplementation or pharmacological activation of the VDR) may help to abrogate AMP deficiency in AD. However, while vitamin D clearly has biological relevance in modulating AMP expression and Th2‐driven inflammation [26, 29], randomized controlled trials and meta‐analyses, particularly in children, have reported inconsistent or limited clinical efficacy [31, 32]. In addition, paradoxical effects have been observed since topical vitamin D_3_ and its analogs such as MC903 and calcipotriol are widely used to induce AD‐like lesions in murine models through keratinocyte‐derived TSLP [33], and case reports describe worsening of AD in children treated with calcipotriol [34]. These observations underscore that, although vitamin D has clear mechanistic importance in AMP regulation and immune modulation, its clinical role in AD is complex and context‐dependent and requires further well‐designed studies to clarify its therapeutic potential.

## 3 Effects of AMPs on the Skin Microbiota and *S. aureus* Colonization in AD

### 3.1 Role of AMPs in Regulating the Skin Microbiota

AMPs serve as key regulators of the skin microbiota, maintaining a balanced microbial ecosystem that protects against pathogenic overgrowth while supporting beneficial commensal populations. AMPs such as hBDs, LL‐37, and S100A7 are constitutively expressed or rapidly induced in response to microbial invasion, whereby they directly inhibit the growth of bacteria, fungi, and viruses [35]. This defensive mechanism is crucial for preventing skin infections and sustaining microbial diversity. Importantly, AMPs not only act as direct antimicrobial agents but also influence the composition of skin‐resident microbes. For example, certain strains of CoNS, which are part of the healthy skin microbiota, produce AMPs that selectively target *S. aureus* [15]. These protective CoNS strains are prevalent on healthy skin but are often absent or significantly reduced in AD patients [15], and their abundance is strongly correlated with *S. aureus* colonization, suggesting that AMP production by CoNS is a critical component of microbial defense.

In addition to direct antimicrobial effects, AMPs also interact with bacterial populations to modulate gene expression related to virulence, adhesion, and biofilm formation [35]. By affecting bacterial signaling pathways, AMPs can reduce the expression of genes that promote colonization and pathogenicity, thereby limiting the ability of harmful bacteria to establish persistent infections. Furthermore, AMPs can influence the host immune response by promoting the recruitment and activation of immune cells, thus enhancing local defense against microbial threats [36].

### 3.2 Roles of AMPs in Dysbiosis and *S. aureus* Overgrowth in AD

In AD, impaired AMP expression (especially the expression of LL‐37, hBD‐2, and dermcidin‐1) contributes to disrupted microbial equilibrium. This effect is characterized by a loss of microbial diversity and a marked overrepresentation of *S. aureus* , the abundance of which is correlated with disease severity [5, 37]. A deficiency of AMPs diminishes the ability of the skin to control microbial overgrowth, thereby allowing *S. aureus* to dominate and trigger inflammation. This microbial imbalance is further exacerbated by structural defects in the skin barrier. For example, mutations in filaggrin (which are common in AD) lead to increased permeability, thus facilitating *S. aureus* adherence and colonization [37]. Once established, *S. aureus* produces virulence factors such as protein A and superantigens, which activate innate immune receptors (such as TLR2) on keratinocytes, thereby inducing proinflammatory cytokines and amplifying Th2‐skewed immune responses [37, 38]. This scenario fosters a chronic inflammatory environment that further suppresses AMP expression, thus creating a self‐reinforcing loop of barrier damage, inflammation, and microbial persistence. Moreover, some pathogenic bacteria, including *S. aureus* , can evade AMP‐mediated killing through multiple mechanisms, including the secretion of proteases that degrade AMPs, the modification of surface charges to repel cationic peptides, and the formation of biofilms that shield bacteria from host immunity and topical treatments [39, 40]. These adaptive mechanisms enable *S. aureus* to persist on the skin despite AMP activity, thus contributing to a chronic disease state and treatment resistance in AD patients.

### 3.3 Feedback Mechanisms Between Microbial Products and AMP Regulation

**FIGURE 1:**
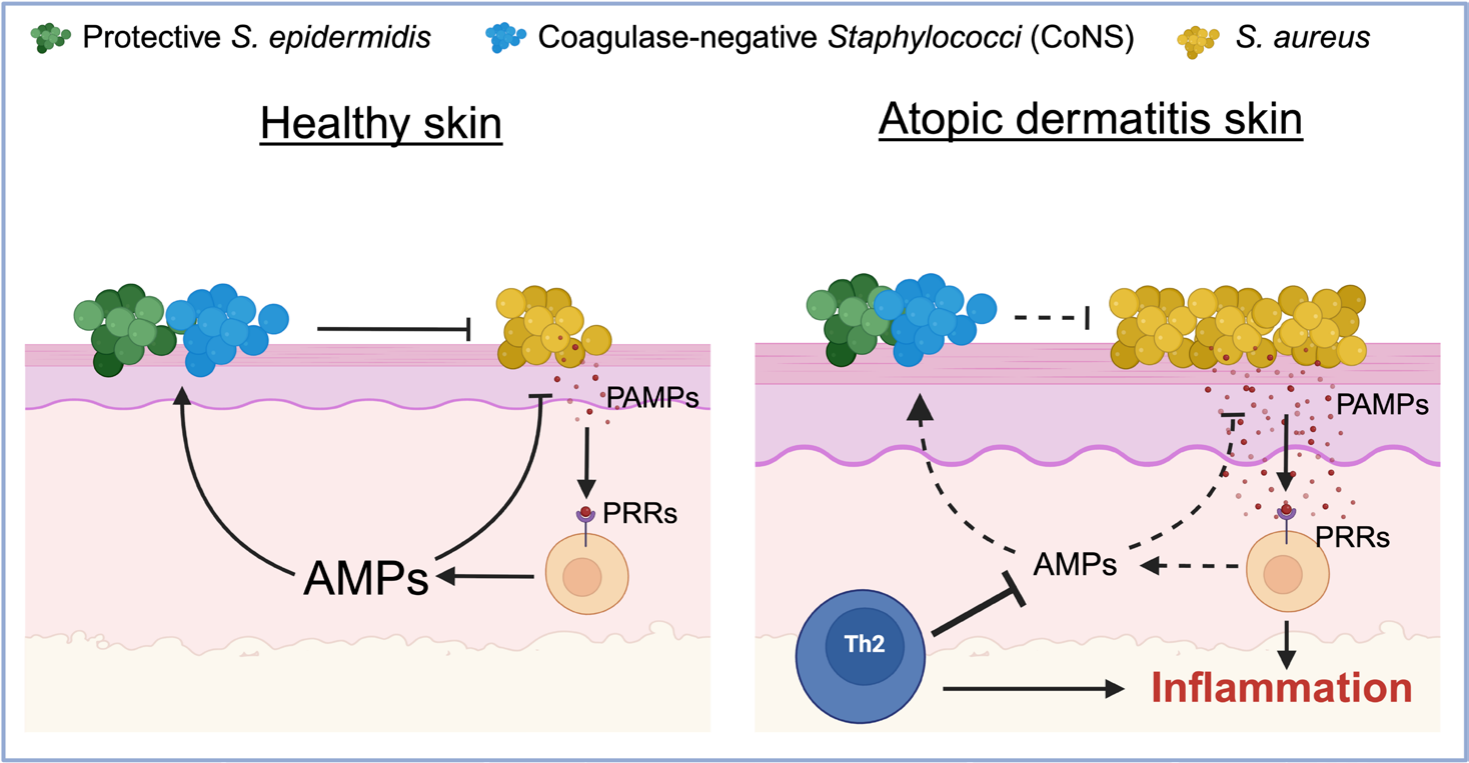
Disrupted AMP–microbiota interactions drive *Staphylococcus aureus* colonization and inflammation in AD. This schematic illustrates the regulatory role of AMPs in maintaining skin microbial balance and their disruption in AD. In healthy skin (left), AMPs such as LL‐37, hBDs, and S100A7 promote the survival of protective *Staphylococcus epidermidis* and other coagulase‐negative staphylococci (CoNS), which inhibit *S. aureus* colonization. Microbial products (PAMPs) stimulate AMP production via pattern recognition receptors (PRRs), thus forming a balanced feedback loop. In AD skin (right), impaired AMP expression permits *S. aureus* overgrowth, which further exacerbates inflammation through the activation of PRRs and the release of virulence factors. Adaptive resistance mechanisms in *S. aureus* (such as protease secretion and biofilm formation) reinforce this dysbiosis–inflammation cycle, thus contributing to chronic disease progression.

The interaction between AMPs and the skin microbiota is bidirectional and dynamically regulated. Microbial products such as lipopolysaccharides and lipoteichoic acid (which are collectively known as PAMPs) activate PRRs such as TLRs and NOD‐like receptors on keratinocytes and immune cells. This activation initiates downstream signaling that induces AMP expression and enhances the antimicrobial barrier of the skin [41]. Conversely, the action of AMPs on microbial populations influences the composition and behavior of the microbiota. When AMPs reduce the microbial load or alter microbial gene expression, the presence of PAMPs is diminished, thereby potentially reducing further AMP induction [35]. In a healthy system, this feedback ensures a balance between microbial control and immune activation. However, in AD, this feedback loop becomes dysfunctional. Th2‐dominant inflammation downregulates AMP expression, thus weakening initial microbial control. In response, *S. aureus* thrives and releases more PAMPs, which continue to activate proinflammatory pathways. Moreover, bacterial resistance mechanisms limit the effectiveness of AMPs, thereby sustaining inflammation and microbial imbalance. An understanding of this disrupted feedback network (Figure 1) is essential for developing new therapeutic strategies. Approaches that increase AMP expression or function (either by supporting beneficial microbes or targeting *S. aureus* virulence) may help to re‐establish microbial balance and disrupt the cycle of inflammation and colonization in AD.

## 4 Immunomodulatory and Barrier‐Related Functions of AMPs in AD

### 4.1 AMPs as Modulators of Inflammation and Cytokine Networks

In addition to their antimicrobial activity, AMPs play a multifaceted role in regulating immune responses in the skin. Depending on their concentration and the local environment, AMPs can exhibit both proinflammatory and anti‐inflammatory properties [42, 43]. At low concentrations, AMPs may enhance adaptive immune responses and promote immune cell migration, whereas at relatively high concentrations, they can directly influence the production of cytokines and chemokines, thus shaping local inflammation. For example, LL‐37 can stimulate the release of cytokines such as IL‐31 from mast cells, thereby contributing to the itching and inflammation that are common in AD [44]. Conversely, some AMPs exhibit anti‐inflammatory properties by dampening cytokine responses to microbial stimuli. For example, the scorpion‐derived peptides known as ToAP3 and ToAP4 reduce TNF‐α and IL‐1β levels in immune cells, thus demonstrating the immunosuppressive potential of specific AMPs in inflammatory conditions [45]. Additionally, hBD‐3 has been reported to suppress AD‐like skin inflammation via aryl hydrocarbon receptor (AhR)‐dependent autophagy induction, suggesting that certain AMPs may attenuate chronic inflammation through regulatory circuits involving barrier and immune signaling [46]. These findings position AMPs not as static defense molecules but as dynamic immunomodulators that are capable of driving the immune balance toward either escalation or resolution, depending on the context and regulation.

### 4.2 Effects of AMPs on Keratinocyte Function and Epidermal Barrier Integrity

Keratinocytes are central to maintaining epidermal barrier function, and AMPs directly influence their behavior by promoting cell proliferation, migration, and differentiation, which are essential for tissue repair and barrier restoration following injury or inflammation [10, 42]. For example, α‐ionone, which is a plant‐derived compound that upregulates hBD‐2 expression, has been shown to accelerate epidermal regeneration by increasing keratinocyte activity [47]. Moreover, AMPs such as LL‐37 and hBD‐3 can modulate the expression of key structural proteins that are vital for skin integrity, such as filaggrin, loricrin, and tight junction components, which are commonly downregulated under the influence of Th2 cytokines in AD [46, 48]. Thus, AMPs contribute not only to microbial defense but also to the structural and functional maintenance of the skin barrier. Their dysregulation in AD may exacerbate both inflammation and barrier dysfunction, thus underscoring their therapeutic relevance.

### 4.3 Dual Roles of AMPs: Protective Versus Pathogenic Functions

AMPs function as a double‐edged sword in AD, where their protective effects can shift toward pathogenic effects depending on their expression levels, proteolytic processing, and local immune contexts. AMPs such as LL‐37 and hBDs provide essential defenses against microbial invasion and help in coordinating the immune response by recruiting immune cells and promoting beneficial cytokine production [7, 49, 50]. Additionally, AMPs such as catestatin and AMP‐IBP5 have been demonstrated to suppress dermatitis‐like symptoms via Notch1 and LRP1 receptor signaling, thereby highlighting their therapeutic potential [36, 51]. Conversely, when AMP production is dysregulated (as is often the case in AD), the protective role of AMPs may be compromised or even reversed. For example, in rosacea, LL‐37 is proteolytically processed into proinflammatory fragments that contribute to disease pathology [49]. A similar mechanism could function in AD under certain enzymatic conditions. Furthermore, excessive AMP levels or altered processing may cause cytotoxicity in host cells and induce hyperinflammation, thus undermining tissue integrity. Therefore, an understanding of the nuanced biology of AMPs is essential. Although the restoration of AMP levels may enhance microbial defense and barrier repair in AD, overactivation can trigger inflammation or tissue damage. These findings underscore that AMP‐based therapeutic strategies must carefully balance protective and pathogenic potential to avoid unintended disease exacerbation.

## 5 Genetic, Epigenetic and Environmental Control of AMPs in AD

### 5.1 Genetic Variants in AMP‐Encoding Genes in AD

Although direct associations between specific AMP gene variants and AD in humans remain under investigation, insights from other species suggest that genetic diversity in AMP‐encoding genes can impact antimicrobial activity. For example, variation in β‐defensin genes in passerine birds has been demonstrated to affect the antimicrobial properties of these peptides [52]. In humans, mutations in skin barrier‐related genes (such as *filaggrin*) are well‐established risk factors for AD [37]. As skin barrier function and AMP expression are closely interrelated, *filaggrin* mutations may indirectly impair AMP‐mediated defenses by increasing skin permeability and susceptibility to microbial invasion. Additionally, polymorphisms or functional variants in genes encoding proteins involved in AMP synthesis (such as proteases and transcription factors) may influence AMP expression or activity in AD [49], although these pathways remain to be fully elucidated. A better understanding of these genetic factors may help to identify individuals at greater risk for AMP‐related immune dysregulation and support the development of targeted or personalized treatment strategies that modulate AMP pathways.

### 5.2 Epigenetic Modifications Regulating AMP Expression in AD

Epigenetic mechanisms (particularly DNA methylation and histone modifications) play key roles in regulating AMP gene expression in AD. Aberrant DNA methylation can silence AMP‐encoding genes, thereby reducing the antimicrobial capacity of the skin. For example, promoter hypermethylation of the *hBD‐1* gene has been identified as a mechanism contributing to its reduced expression in AD lesional skin [53]. Although histone modifications in the context of AMP regulation in AD have been less studied, their role is likely significant. Changes in histone acetylation or methylation can alter chromatin accessibility and modulate the transcription of AMP‐related genes [53]. Characteristic inflammatory signals of the AD environment may influence these epigenetic marks and further suppress AMP production. Moreover, therapeutic interventions may influence AMP expression through epigenetic pathways. Narrowband ultraviolet B phototherapy, which is commonly used in AD treatment, has been demonstrated to increase cathelicidin levels and decrease hBD‐2 expression in healing lesions [54]. These changes may be mediated via vitamin D signaling and shifts in the cytokine milieu, thus potentially involving epigenetic remodeling of AMP gene loci.

### 5.3 Environmental and Pharmacological Modulators of AMP Expression in AD

Environmental exposures and pharmacological agents can significantly modulate AMP expression and function in AD, either by exacerbating or alleviating disease severity. One of the most well‐studied factors is vitamin D, which influences both innate and adaptive immune responses. Low serum vitamin D levels are associated with increased AD incidence and severity [26], whereas supplementation with vitamin D potentially reduces clinical symptoms by normalizing the Th1/Th2 cytokine balance and increasing AMP production [30]. The detailed mechanism by which vitamin D modulates AMP production is described in Section 2.2.

AhR, which is a ligand‐activated transcription factor that is responsive to environmental pollutants and certain skin treatments, also plays a key regulatory role in skin inflammation and AMP expression in keratinocytes. Although sustained AhR activation leads to AD‐like phenotypes in transgenic mice [55], AhR activation by coal tar in keratinocytes can also induce the expression of several AMPs, thereby contributing to the restoration of skin microbial balance and barrier function [56]. These findings underscore the dual nature of environmental signals in modulating AMP expression (involving either the promotion of disease or aid with recovery), which is dependent on the context and duration of exposure.

## 6 Therapeutic Implications and Future Directions for AMPs in AD

### 6.1 AMP‐Based and AMP‐Enhancing Therapies

Therapeutic strategies targeting AMPs exhibit considerable promise for treating AD, in which AMP dysregulation contributes to microbial overgrowth and immune imbalance. One approach involves the development of synthetic AMPs with improved properties (including enhanced stability, a broader antimicrobial spectrum, reduced toxicity, and better resistance to proteolytic degradation) [57]. These engineered peptides can be tailored to target specific pathogens or modulate immune responses in a controlled manner. Another strategy involves increasing AMP production in the skin. Certain compounds, such as coal tar and tapinarof, can stimulate endogenous AMP expression via the activation of signaling pathways such as the AhR pathway [56, 58]. This mechanism helps to restore microbial balance and reduce inflammation in the skin. Natural compounds, vitamin D analogs, and AhR agonists are currently being investigated as AMP‐enhancing agents. Although they are still considered to be experimental treatments, gene therapy approaches that correct genetic defects impairing AMP production could eventually offer personalized interventions for select AD patients.

### 6.2 Balancing the Antimicrobial and Inflammatory Properties of AMPs

Achieving a balance between the antimicrobial and inflammatory properties of AMPs is crucial for their effective use in the treatment of AD. In AD, there is a delicate balance between the need to control microbial infections (which requires the antimicrobial activity of AMPs) and the need to reduce inflammation, as excessive inflammation can exacerbate the disease [59]. Some AMPs have been shown to possess both antimicrobial and anti‐inflammatory properties. For example, the peptide D‐Mt6, which is an analog of MAF‐1, not only demonstrates broad‐spectrum antimicrobial activity against *Acinetobacter baumannii* but also exhibits anti‐inflammatory effects by decreasing the secretion of proinflammatory cytokines such as IL‐1β and TNF‐α in LPS‐stimulated macrophages [60]. To achieve optimal balance, the design and development of AMP‐based therapies need to consider the concentration‐dependent effects of AMPs. Specifically, at low concentrations, AMPs may primarily exert immunomodulatory effects, whereas at higher concentrations, their antimicrobial activity may dominate. Additionally, the use of delivery systems can help to control the release and distribution of AMPs, thereby ensuring that they act at the appropriate site and concentration to balance their antimicrobial and inflammatory properties. Moreover, an understanding of the mechanisms by which AMPs perform these dual functions is essential for the development of more effective AMP‐based therapies for AD.

### 6.3 Challenges and Translational Prospects of AMPs in AD

Despite the promising potential of AMPs in the treatment of AD, several challenges need to be addressed for their successful translation into clinical practice. One of the major challenges involves the cytotoxicity of AMPs. Some AMPs may have toxic effects on host cells, which can limit their use as therapeutic agents. For example, certain AMPs may cause hemolysis or damage to keratinocytes, which can worsen the skin conditions [18]. Strategies to reduce the cytotoxicity of AMPs, such as chemical modifications or the use of delivery systems to specifically target AMPs to the site of infection, need to be further explored. Another challenge involves the susceptibility of AMPs to proteolytic degradation. In the skin environment, proteases can elicit the breakdown of AMPs, thus reducing their effectiveness. The development of AMPs that are more resistant to proteolysis or the use of protease inhibitors in combination with AMPs could be potential solutions for this issue. Additionally, the high costs of production and the lack of a clear understanding of the long‐term safety and efficacy of AMP‐based therapies are also barriers to their widespread use. However, with the continuous advancement of technology, such as the development of more efficient peptide synthesis methods and improvements in delivery systems, the translational prospects of AMPs in AD are promising. Future research should focus on overcoming these challenges to fully understand the potential of AMPs in the treatment of AD.

## 7 Conclusion

AMPs represent a crucial link between innate immunity, microbial homeostasis, and skin barrier integrity. In the context of AD (a condition characterized by disrupted barrier function and dysregulated immune responses), AMPs play a dualistic role. For example, their antimicrobial and immunomodulatory activities offer protection against pathogens such as *S. aureus* and help shape the local immune environment. In contrast, their reduced expression or aberrant regulation (which is largely driven by Th2 cytokines such as IL‐4 and IL‐13) undermines these protective functions and contributes to microbial dysbiosis, inflammation, and barrier dysfunction. Recent studies have further illuminated the complex signaling networks through which AMPs interact with keratinocytes, immune cells, and microbial communities. Mechanistic insights into pathways such as vitamin D/VDR signaling, autophagy, and AhR activation have provided new therapeutic possibilities. AMP mimetics, synthetic peptides, and interventions targeting AMP‐modulating pathways (such as vitamin D analogs or AhR agonists) are emerging as promising strategies for restoring barrier integrity and immunological balance in AD. Overall, AMPs are not merely effector molecules in host defense; rather, they are dynamic regulators of skin physiology. An understanding of their context‐dependent functions is essential for designing precise therapeutic interventions that harness their benefits while avoiding potential proinflammatory or cytotoxic effects. Future research should aim to delineate AMP subtypes with therapeutic potential, define optimal delivery systems, and clarify their role in the crosstalk between the immune system, skin barrier, and microbiota in AD and related inflammatory skin disorders.

## Conflicts of Interest

The authors declare no conflicts of interest.

## Data Availability

Data sharing not applicable to this article as no datasets were generated or analyzed during the current study.
